# Is Social Media a Boon or Bane for Orthodontics in the Current Digital Age: A Cross-Sectional Electronic Survey

**DOI:** 10.7759/cureus.78164

**Published:** 2025-01-28

**Authors:** Tabassum Qureshi, Kaleem Fatima, Karthik Sennimalai, Om P Kharbanda

**Affiliations:** 1 Orthodontics, Jamia Millia Islamia, Delhi, IND; 2 Orthodontics and Dentofacial Orthopaedics, Maulana Azad Institute of Dental Sciences, New Delhi, IND; 3 Orthodontics, All India Institute of Medical Sciences, Jammu, Jammu, IND; 4 Orthodontics and Dentofacial Orthopaedics, Ramaiah University of Applied Sciences, Bengaluru, IND

**Keywords:** cross-sectional survey, #instagram, orthodontics, questionnaire, social media

## Abstract

Background: Social media is an online platform where people share their experiences, perspectives, and opinions individually or in groups. There has been a rapid increase in internet users after the introduction of social media sites such as Facebook, YouTube, Instagram, and Twitter. Other than primary means of communication, social media aids in distributing healthcare information among professionals and patients. It enhances the knowledge of health care to the patients, but on the contrary, patients can be misguided by false information influenced by unauthorized professionals as it is readily available. With the same objective in mind, the study was undertaken to investigate the perspective of Indian orthodontists on social media use and to assess the potential benefits and disadvantages of social media along with its role in health care education.

Materials and methods: A cross-sectional online questionnaire survey was designed to consist of 42 questions related to the perspectives of Indian orthodontic professionals on the use of social media in orthodontic practice. The questionnaire was sent to 800 registered Indian orthodontists through electronic mail.

Results: A total of only 173 orthodontists responded to the online survey. The electronic survey showed that the phone was the most preferred communication tool in the practice with the patients (87.2%). The most common social media platform for sharing patient information was Instagram (82.5%). The survey showed that none of the orthodontists have received formal training or certification on using social media (85.7%). The reliability of social media platforms is very low (34.9%). Social media would affect the patient's choice of healthcare provider (87.3%). The majority of orthodontists have agreed that there should be regulations for quality control on social media to share patient information (90.5%).

Conclusions: The study's findings offer valuable insights into the current landscape of social media usage among Indian orthodontists. While social media presents numerous opportunities for communication, education, and practice promotion, it also poses challenges related to training, misinformation, patient privacy, and regulatory oversight. Addressing these challenges requires a concerted effort from orthodontists, regulatory bodies, and digital platforms to develop and implement guidelines that promote responsible social media usage while upholding professional standards and patient welfare. By leveraging social media effectively and ethically, orthodontists can enhance patient engagement, education, and practice visibility in the digital age.

## Introduction

The Internet has revolutionized communication from traditional paper-based to modern smartphone-based applications. Social media is an online platform where people share their experiences, perspectives, and opinions individually or in groups. It has been the fastest and cheapest way of transferring messages globally. It has been estimated that there are more than 4.5 billion internet users after a rapid increase in smartphone usage worldwide [1,2]. There has been a rapid increase in internet users from 8% to 72% since 2005 in the United States after the introduction of social media sites such as Facebook, YouTube, Instagram, and Twitter [3]. Other than primary means of communication, social media aids in distributing healthcare information among professionals and patients. It enhances the knowledge of health care to the patients, but on the contrary, patients can be misguided by false information influenced by unauthorized professionals as it is readily available [4].

Similar trends are seen in orthodontics, where most patients are young adolescents actively involved in social media sites. These platforms have become a valuable source of information, providing quality content on orthodontic awareness [5], retention [6], fixed braces [7], and oral appliance therapy [8]. In this digital era, the use of updated 3D digital technologies has significantly improved orthodontist skills, leading to better patient care and increased patient engagement. When shared on social media, these updates go viral among peer groups, further enhancing orthodontic education [5]. Additionally, social media is used as a tool for social marketing, providing a platform to promote services and products [9]. Before the advent of the Internet and social media platforms, patients would typically seek the services of reputable orthodontists either by directly visiting their offices or obtaining referrals. Today, in the digital world, websites offer instant access to information on orthodontists and their treatments. Thus, social media platforms are implemented as potent tools to enhance visibility [10]. Uploading photos and videos of patients and their reviews using hashtags has been a recent trend to communicate with potential patients [11]. Unfortunately, cybercrime involving digital content theft is prevalent on online sites, often prompted by those seeking false individual credibility and financial gains. Patients may sometimes be unaware that their information is being updated online, which is considered unethical [12]. Furthermore, the paid promotions and increased number of followers for consumer engagement could misguide patients if the information is not authentic [13].

Social media and telehealth sites have been used as sources of health care information, training professionals, and providing clinical and supervision services. It also provides accessibility to socially isolated individuals who lack meaningful community engagement and participation [12]. There has been limited evidence on the effectiveness of social media sites regarding healthcare information, marketing, and ethical concerns regarding patient digital data. Therefore, this study aimed to evaluate the perspective of Indian orthodontists on the digital content of orthodontic treatment on social media using validated questionnaires. In addition, guidelines should be developed for posting content of orthodontic patient-related videos online.

## Materials and methods

A cross-sectional online questionnaire study was conducted among orthodontists practicing across different parts of India. Ethical approval was obtained from the institutional ethical committee. (31/1/480/JMI/IEC/2024). The sample size was calculated before the start of the study using a 95% level of confidence, 5% precision, and 50% prevalence, which led to a sample size of 385 participants [7]. Considering the attrition rate of 50% and non-responders, a total sample size of 800 was estimated. A pre-validated questionnaire was developed in Google Forms [21] and was sent individually to all the practitioners through WhatsApp and electronic mail. The aim and objective were briefed in the form before the start of the survey, and all participants were reminded three times. Informed consent was obtained before the start of the survey. Registered orthodontic practitioners with more than one year of experience, irrespective of gender or age, were the only ones included in this survey. In case of non-response, the participants were excluded from the study.

Questionnaire development

A set of questionnaires related to social media and its application in orthodontic practice was formulated by two senior orthodontists. The validation was done by sending a set of questionnaires to a panel of eight senior orthodontists with more than twenty years of experience who were not part of this study. 1The structured questions were derived from expert opinion, research evidence, and existing questionnaires. The experts administered a content validation of drafted questions based on the four-point scale: 4 = highly relevant, 3 = quite relevant, 2 = somewhat relevant but needs rewording, and 1 = not relevant. Finally, a set of 42 items that were highly relevant and quite relevant were included in this study and presented in clear and comprehensible language. All the questions were in the English language only. The formulated questions were later sent to 20 other orthodontists and re-examined after one month for inter-rater reliability, and a good agreement was reached of 0.88. The final 42 questions were in the editable Google form outlined in the Supplementary file. The set of questions was derived from expert opinion, literature reviews, and existing research on social media's role in healthcare communication. Additionally, the questions were validated through content validation (using a four-point scale) by a panel of senior orthodontists, and the final set was tested for inter-rater reliability [5], and a good agreement was reached of 0.88. The final 42 questions were in the editable Google form outlined in the Supplementary file.

Questionnaire design

The set of 42 questionnaires was formulated in five sections, and section one was informed consent. Section two, from questions two to 14, was related to demographic details of practicing orthodontists. It also included the preferred communication tool or social media platform. The section involves the preference and frequency of usage of social media platforms for sharing patient information, patient care, and marketing. There are ethical concerns regarding posting photos and videos of orthodontic patients. It also involves practitioners' formal training and manpower to handle social media platforms. If the patient is not using social media to share patient information, the participants can skip section three. Section four questions from 34 to 42 describe the practitioner's disinterest, disadvantage, reliability, and regulatory management of social media platforms. The final section expresses gratitude and appreciation for the response of participants. Descriptive statistical analysis was performed for the analysis of data.

## Results

**Figure 1:**
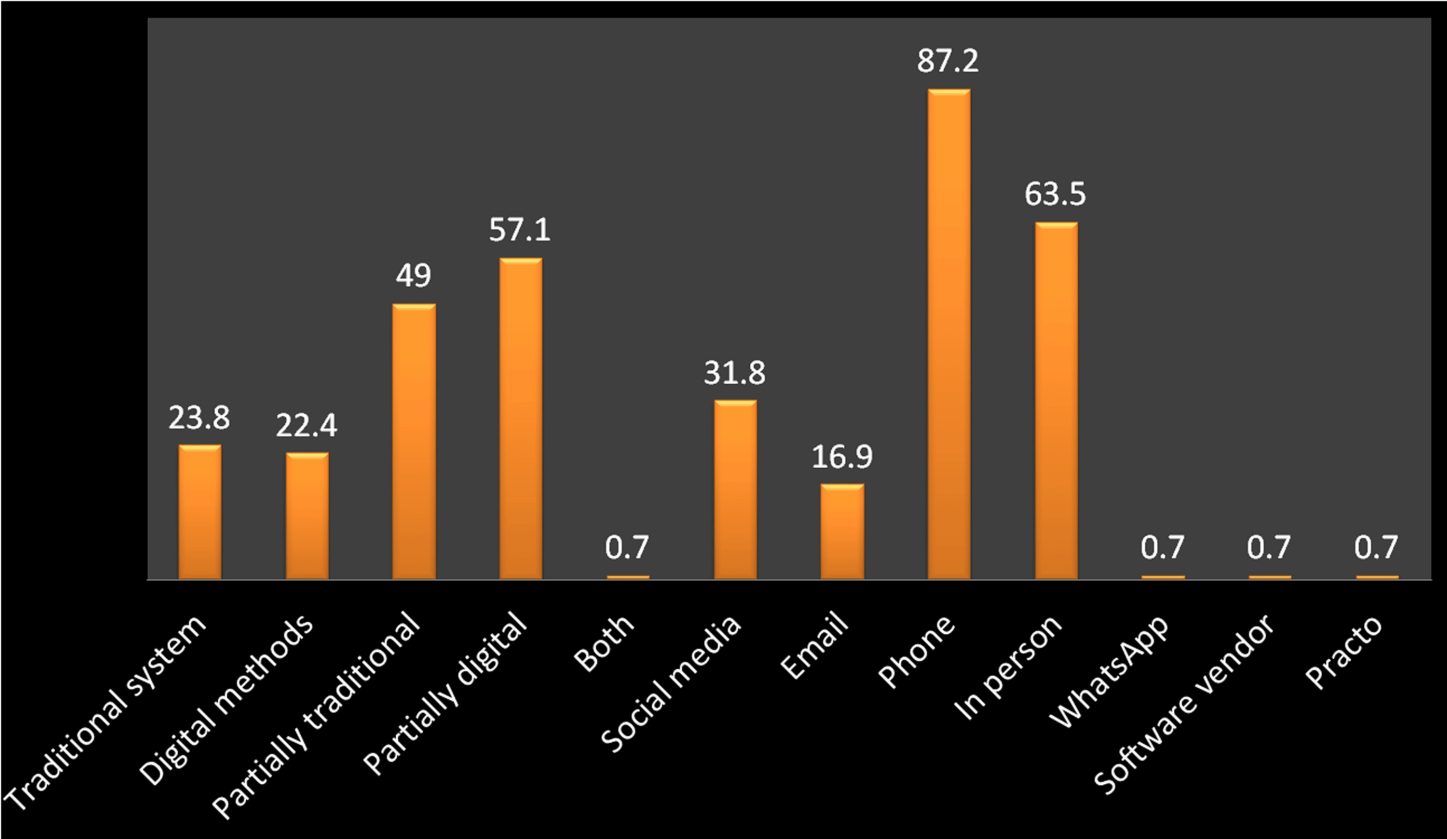
Mode of connecting with patients during orthodontic practice (%)

A total of 800 orthodontists across India were invited to participate in this online questionnaire survey. Only 173 participants voluntarily agreed and responded, constituting approximately 21.5%. The age group of participants ranged from 27 to 76 years; among them, 58% were male participants. Most of them were clinicians (68%), followed by academicians affiliated with institutions (40%). In addition, many of them reside in urban cities, and it was also found that orthodontic records were partially handled through digital methods (Figure 1).

**Figure 2:**
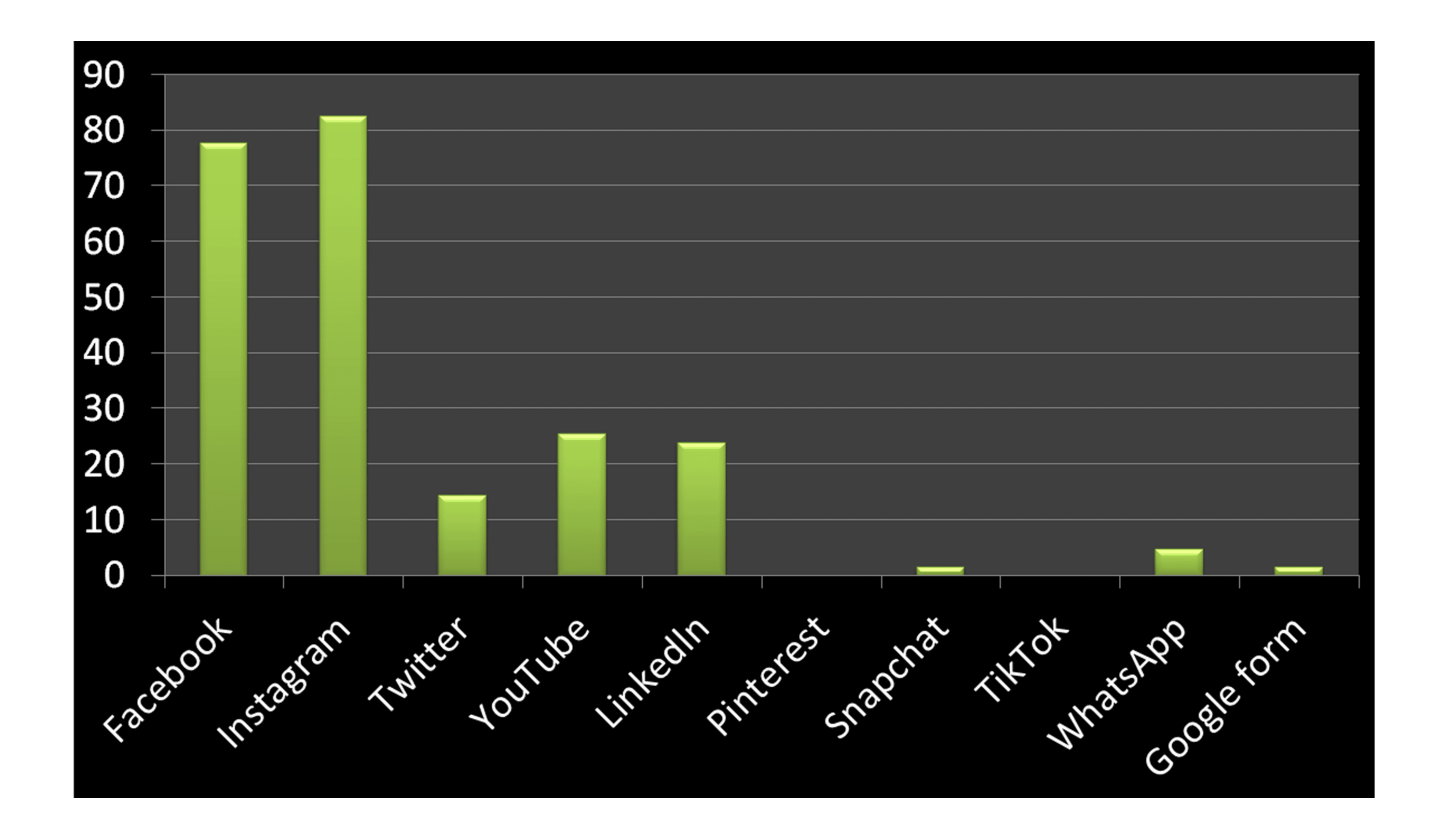
Type of social media platform use during orthodontic practice (%)

The survey revealed that 87.2% of orthodontists use phones as their preferred communication tool, and only 32% use social media (Figure 1). Despite lesser usage of social media, 82.5% of orthodontists utilize Instagram (California, USA), while 77.8% utilize Facebook (California, USA) to share patient-related information on social media platforms (Figure 2). Out of the 63 orthodontists surveyed, 85.7% acknowledged not receiving any formal training on social media usage. Additionally, 69.8% stated they lack dedicated manpower to manage their social media profiles (Figure 2). 

**Figure 3:**
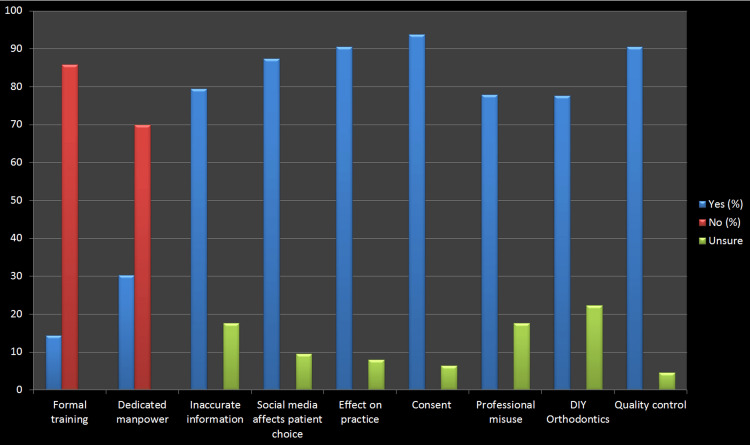
The response of various questionnaires

It was believed that most of the orthodontists (39.9%) questioned the reliability of information available on social media platforms. Furthermore, 87.3% of orthodontists reported that social media potentially influences decisions made by patients and also selecting healthcare providers. Therefore, 79.4% of the participants believed that it's the responsibility of professionals to denounce inappropriate and inaccurate orthodontic health-related information surfacing online. Soon, social media will continue to rise as a marketing tool, as suggested by 95.5% of the orthodontists in the survey (Figure 3). 

One of the important factors regarding ethical concerns is posting patient records online. The majority of orthodontists (93.7%) took consent from patients before posting on social media. Unfortunately, the misuse of records was not under the control of the trained professionals. In addition, 74.6% of orthodontists believed that patients are unaware of the disadvantages of self-diagnosis and do-it-yourself (DIY) surfacing online. Therefore, 90.5% of orthodontists emphasized the need for regulations of patient-related health information posted on different social media platforms (Figure 3).

Respect for patient confidentiality when publicizing patient-related information on social media was considered paramount by many surveyed orthodontists (24%). In addition, the information that is shared on social media should be authentic and evidence-based, as highlighted by most orthodontists (61%). These important regulatory considerations were believed to ensure professional orthodontists' ethical responsibility and credibility without data manipulation. Therefore, standardized regulations should govern the authenticity of online uploading of patients' treatment records, focusing mainly on evidence-based practices and treatment mechanics supported by literature. The senior orthodontist or any higher regulatory body should verify the content. 

**Figure 4:**
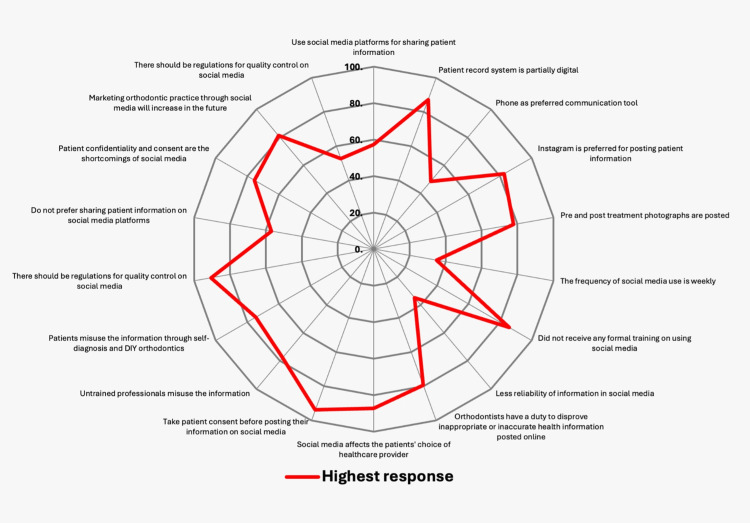
The highest response of the questionnaire during the social media survey

The other concerns raised by the professionals in the survey were the need for regulations for DIY orthodontics and unauthorized orthodontic practice by normal dentists who did not complete the orthodontic residency program. The Dental Council of India (DCI) and the Indian Orthodontic Society (IOS) should strictly warn those who post misleading and unscientific patient health care-related information. It is advisable to avoid posting clinical videos of procedures that could be misused. Most importantly, misguiding the patients with faster treatment with only six social corrections by untrained professionals and marketing appliances without any scientific evidence was important. Therefore, undue fake promises by corporate chains should be strictly stopped, and strict action should be taken against them. The important questionnaires related to the use of social media as orthodontic healthcare information with their responses are compiled in Table 1, and the diagrammatical representation of the highest response is depicted in Figure 4.

**Table 1:** The important social media related questionnaire

Questionnaire	Highest response	Least response
What method of the patient record system do you use in your orthodontic practice?	Partially digital (57.4%)	Digital method (22.3%)
What is the preferred communication tool in your practice with patients?	Phone (87.2%)	WhatsApp (0.7%)
Do you use social media platforms for sharing patient information?	Yes (48.5%)	No (51.5%)
Which social media platform(s) do you prefer to post information and pictures related to orthodontic treatment?	Instagram (82.5%)	TikTok (0.0%)
What types of content do you post on social media platforms?	Pre and post treatment photographs (77.8%)	Update of clinical gadgets and Testimonials (1.6%)
How frequently do you use social media related to orthodontic practice?	Weekly (34.9%)	Rarely (17.5%)
Have you ever received any formal training or certificate on using social media?	No (85.7%)	Yes (14.3%)
How reliable do you find information on social media?	34.9%	
Do orthodontists have a duty to disprove inappropriate or inaccurate health information posted online?	Yes (79.4%)	Unsure (17.5%)
Do you think social media would affect the patients' choice of healthcare provider?	Yes (87.3)	Unsure (9.5%)
Do you take consent from your patient before posting their information on social media?	Yes (93.7)	No (6.3%)
Do you feel untrained professionals misuse the information available on social media to treat orthodontic patients?	Yes (77.8%)	Unsure (17.5%)
Do you feel patients misuse the information available on social media through self-diagnosis and DIY orthodontics?	Yes (74.6%)	Unsure (22.2%)
Do you think there should be regulations for quality control on social media for the purpose of sharing patient information?	Yes (90.5%)	Unsure (9.5%)
Why would you not prefer sharing patient information on social media platforms?	Security concerns (57%)	Ethical (1%)
What are the shortcomings of social media according to you?	Patient confidentiality and consent (75.6%)	Time: benefit ratio (1.2%)
Do you think that marketing orthodontic practice through social media will increase among orthodontists in the future?	Yes (81.2%)	Unsure (12.9)

## Discussion

The orthodontic field has undergone a significant transformation in recent years as a result of the pervasive adoption of social media platforms. These platforms have become indispensable resources for orthodontists, allowing them to establish connections with patients, disseminate information regarding treatment alternatives, and exhibit their work to a broader audience. Nevertheless, there are apprehensions about the influence of social media on orthodontic practice, despite the numerous benefits [10]. Therefore our study assessed the perspectives of Indian orthodontists on the use of social media in their practice, providing a comprehensive understanding of a variety of factors, including usage patterns, training, regulatory considerations, and concerns.

A wide range of respondents aged from 27 to 76 years among 173 voluntary participants in this survey. The diverse clinical experiences among the participants varied the perspectives on access to social media. Young orthodontists preferred the use of social media, and there was a steady decrease with age. This is due to awareness and ease of accessibility of the Internet among young orthodontists. This aligned with the results of our study. Many prefer the use of social media platforms for health care information and patient awareness; unfortunately, very few use them as tools. The term "tools" refers here to electronic patient monitoring systems that leverage social media for activities such as interacting with patients, providing health information, managing a professional online presence, or engaging in marketing efforts in a systematic and responsible way. There are multiple software programs for electronic patient monitoring systems. Similarly, corroborating with other studies, Facebook and Instagram were preferred platforms among Indian orthodontists for uploading or sharing patient-related information [14]. 

Despite the relatively low adoption rate of social media overall, these findings highlight the popularity of specific platforms among orthodontists for professional purposes. A concerning revelation from the study was the lack of formal training in social media usage among orthodontists, with 85.7% admitting to not receiving any training. Additionally, nearly 70% of respondents stated that they lacked dedicated manpower to manage their social media profiles effectively. This underscores the need for educational initiatives and support systems to equip orthodontists with the necessary skills and resources to navigate social media platforms responsibly.

The prevalence of concerns regarding the reliability of information on social media platforms and the potential influence on patient decisions echoes findings from previous studies. Almost 40% of respondents acknowledged the unreliability of information on social media platforms, highlighting the prevalence of misinformation and its potential impact on patient care. Additionally, a significant majority (79.4%) felt a professional obligation to refute inappropriate or inaccurate health-related content disseminated online. This concern is justified, as inaccurate information can lead to unrealistic expectations, self-diagnosis attempts, and potentially harmful DIY treatments [15]. Furthermore, the study highlighted orthodontists' concerns about the influence of social media on patient decision-making. Most respondents (87.3%) agreed that social media could sway patients' choices when selecting healthcare providers. These findings resonate with previous research, highlighting the need for orthodontists to address patient expectations and concerns filled by social media content [16].

Despite the concerns, the study acknowledges the potential benefits of social media. A significant majority (87.3%) believe social media influences patient choice of healthcare providers. This aligns with broader trends in healthcare communication, where patients increasingly rely on online resources for information and decision-making. Additionally, nearly all orthodontists (95.5%) anticipate increased use of social media marketing in the future. This implies that social media can be an influential tool for the propagation of practices, patient education, and the cultivation of a more robust online presence. Therefore, the advantages and disadvantages of social media's influence in orthodontics must be understood well [5].

The overwhelming support for regulations (90.5%) suggests a desire for clear guidelines prioritizing patient well-being and ethical practices. The proposed regulations, emphasizing patient confidentiality, evidence-based information, and ethical case sharing, provide a valuable framework. Banning unregistered practices and DIY treatments directly addresses the risks associated with misleading information [17]. Furthermore, standardizing case presentations can ensure transparency and discourage unrealistic promises. These suggestions echo concerns raised in other studies highlighting the need for professional bodies to establish clear guidelines for social media use in orthodontics [18].

Regulatory considerations emerged as a significant theme in the study, with 90.5% of orthodontists emphasizing the need for regulations to ensure quality control when sharing patient-related information on social media platforms. Specific regulatory suggestions included maintaining patient confidentiality, ensuring shared information's authenticity and evidence-based nature, and standardizing practices to uphold professionalism and ethics [19]. Moving forward, regulatory bodies and professional associations must develop comprehensive guidelines and best practices for social media usage in orthodontic practice. Collaboration between orthodontists, regulatory bodies, and digital platforms is crucial to promote responsible social media usage while safeguarding patient welfare and upholding professional standards [20]. The various software available for patient tracking and monitoring are electronic health record (EHR) software, medical database software, medical research software, medical diagnostic software, telemedicine software, appointment scheduling software, health tracking apps, and remote patient monitoring. These software programs became essential tools wherein data-driven practices revolutionized the healthcare system. It helps in interacting with patients, providing health information, managing a professional online presence, or engaging in marketing efforts in a systematic and responsible way.

Limitations

The study's relatively low response rate (21.5%) necessitates caution when generalizing the findings to the entire population of Indian orthodontists. Further research with a larger and more representative sample could provide a more comprehensive picture. Additionally, exploring patients' perspectives through surveys and focus groups could offer valuable insights into their online behavior and how they utilize social media to find information about orthodontists. Considering both the provider and patient perspectives, this two-pronged approach can pave the way for developing effective social media strategies in orthodontics.

Future aims and scope

The future aims and scope of the study are to explore the role of the metaverse, which serves as a platform for orthodontic consultation, patient education, and virtual treatment simulations, enhancing patient understanding and engagement. The study also aims to develop comparative digital platforms where it can assess the effectiveness of traditional social media versus immersive metaverse environments in promoting orthodontic awareness, professional branding, and patient interaction. Virtual orthodontic clinic in the metaverse where patients can interact with orthodontists, visualize treatment outcomes, and access resources in a fully immersive environment. Social media can also be helpful for enhancing professionals training and incorporating virtual reality for learning complex procedures and improving clinical skills. It can improve patient journeys, collaboration, and networking patient journeys from virtual consultations to post-treatment follow-ups, fostering a more engaging and informed process. There is also a scope of digital wellbeing, which helps to understand the psychological impact of immersive technologies like the metaverse on patients and professionals, ensuring a balanced approach to its adoption in orthodontics.

## Conclusions

The study's results provide valuable insights into the current state of social media usage among Indian orthodontists. In addition to the numerous opportunities for communication, education, and practice promotion that social media offers, it also presents challenges in patient privacy, regulatory supervision, misinformation, and training. To confront these obstacles, orthodontists, regulatory bodies, and digital platforms must collaborate in developing and implementing guidelines that prioritize responsible social media usage while maintaining professional standards and patient welfare. Orthodontists can improve patient engagement, education, and practice visibility in the digital age by ethically and effectively utilizing social media.
